# (Alzheimer's) dementia in adults with Down syndrome in Germany: Administrative prevalence based on a claims data analysis

**DOI:** 10.1177/13872877261449417

**Published:** 2026-05-15

**Authors:** Milena Weitzel, Anke Walendzik, Godwin D. Giebel, Pascal Raszke, Jürgen Wasem, Johannes Levin, Georg Nübling, Olivia Wagemann, Elisabeth Wlasich, Johannes Pantel, Valentina A. Tesky, Arthur Schall, Thomas Ruhnke, Patrik Dröge, Theresa Hüer

**Affiliations:** 1 Institute for Health Care Management and Research, 27170 University of Duisburg-Essen, Essen, Germany; 2 Essen Research Institute for Medical Management, EsFoMed GmbH, Essen, Germany; 3 Department of Neurology, LMU University Hospital, 9183 LMU Munich, Munich, Germany; 4 German Centre for Neurodegenerative Diseases, Site Munich, Munich, Germany; 5 Munich Cluster for Systems Neurology (SyNergy), Munich, Germany; 6 Institute for General Practice, Goethe University, Frankfurt am Main, Germany; 7 AOK Research Institut (WIdO), Berlin, Germany

**Keywords:** Alzheimer's disease, dementia, Down syndrome, diagnosis, health care, prevalence

## Abstract

**Background:**

People with Down syndrome (DS) have a significantly increased risk of developing early-onset Alzheimer's disease. For example, a longitudinal study by McCarron et al. (2017) found that 97% of a cohort of 77 DS patients aged 35 years and older developed dementia. Despite this high risk, administrative data on dementia prevalence in this population remain limited.

**Objective:**

This study examines whether the diagnosed prevalence is lower than expected based on epidemiological data and explores differences compared to the general population.

**Methods:**

A comparative analysis of administrative dementia prevalence (2010–2019) was conducted using claims data for adults with and without DS. Prevalence rates were calculated by age and sex. Chi-square tests were applied to assess significance (p < 0.05), with Cramér's V and Phi as measures of association. Odds ratios were calculated to evaluate group differences.

**Results:**

Total administrative dementia prevalence was significantly higher in adults with DS (Mean Value (MV) 9.2% ± 1.7% (Standard Deviation (SD))) than those without DS (MV 3.2% ± 0.3% (SD)). Age- and sex-specific analyses also revealed notable differences. For example, in the 56–60 years age group, prevalence was MV 28.7% ± 4.6% (SD) in adults with DS versus MV 0.7% ± 0.1% (SD) in those without DS.

**Conclusions:**

Although administrative dementia prevalence is higher among adults with DS than those without DS, observed rates appear lower than expected based on existing epidemiological data. This suggests a potential underdiagnosis of dementia in the DS population in Germany.

## Introduction

With a prevalence of 10.1 per 10,000 live births, Down syndrome (DS), also known as trisomy 21, represents the most common chromosomal aberration in Europe.^1^ In recent decades, the life expectancy of adults with DS has increased significantly. While the average life expectancy of this population was 45 years in 1989,^2^ it has now risen to over 60 years.^3^ This is particularly due to medical progress in the treatment of comorbidities such as heart defects.^4^

However, this positive development brings new challenges. Adults with DS have a genetically determined, significantly increased risk of developing Alzheimer's disease (AD) at an early age.^5,6^ This is due to the gene for the Amyloid Precursor Protein, located on chromosome 21, which is present three times in most adults with DS.^7,8^ Amyloid precursor proteins are involved in the formation of the amyloid plaques that are characteristic of AD.^7^ Due to the medical progress in the treatment of other comorbidities, AD has now become the most common cause of death among adults with DS.^9^

The prevalence of (Alzheimer's) dementia in individuals with DS has been the subject of investigation in a number of epidemiological studies.^8,10–15^ In a 20-year-long longitudinal study of 77 DS patients aged 35 years and older, 97% developed (Alzheimer's) dementia.^10^ The estimated onset of the disease in adults with DS is, depending on the study, between 45 and 59 years on average.^8,10,16–18^ As (Alzheimer's) dementia testing procedures were used regularly in these studies, the results can be considered a reliable representation of the actual rates. In comparison, the average age of onset of (Alzheimer's) dementia in the general population is significantly higher, at about 80 years.^19,20^

Research indicates that health inequities exist in the care of people with intellectual disability.^21,22^ Although significant progress has been made over the past decades, adults with DS continue to encounter distinct barriers to the diagnosis of (Alzheimer's) dementia when compared to the general population. For example, standardized test procedures (e.g., the Clock-Drawing Test) are not applicable, unlike in the general population.^23,24^ Although (Alzheimer's) dementia tests specifically developed for people with intellectual disabilities exist (e.g., DSQIID,^25^ the DTIM,^26^ the NTG-EDSD^27^ or the CAMDEX-DS^28^), they are often not well known to standard care providers in Germany. Furthermore, physicians frequently rely on third-party anamnesis provided by caregivers, particularly in the assessment of behavioral changes in patients.^24^ The reliable detection of neurodegenerative changes through MRI may be impeded in individual cases where the procedure is not tolerated by the patient.^24^ Due to these persistent barriers, disparities may exist in the diagnostics provided to people with (Alzheimer's) dementia, depending on whether or not they have DS.

Despite the diagnostic challenges, recognizing dementia in adults with DS remains crucial, as this is the only way to avoid misdiagnosis (which can lead to inappropriate medication, for example), ensure appropriate treatment and to provide better support für those affected and their caregivers.

The aim of this study is to investigate the proportion of adults with DS who receive an (Alzheimer's) dementia diagnosis in standard care. To address this, the study follows a three-step approach. First the study analyzes coded (Alzheimer's) dementia diagnoses in claims data for adults with DS. Second, these results are compared with the epidemiological data described in the introduction section. In a third step, coded (Alzheimer's) dementia is analyzed in a group of adults without DS. This comparison should provide insight into whether any underdiagnosis identified in the DS group is related to the challenges faced by this group or represents an overall underdiagnosis of (Alzheimer's) dementia. This comparison will therefore help distinguish system-level factors from DS-specific factors, such as differences in diagnostic practices or access to specialist care. The study is part of the project “(Access to) Diagnosis and Treatment of Dementia in People with Down Syndrome” (“DS-Demenz”, grant number: 01VSF21030), funded by the Innovation Fund.

## Methods

### Data

For the analysis, we used anonymized health claims data provided by Allgemeine Ortskrankenkasse (AOK, local health care funds) Research Institute (WIdO) of the years 2010 to 2019. The dataset comprised data from eleven legally independent statutory health insurance (SHI) funds of the “AOK—Die Gesundheitskasse” (AOK), which together cover around one third of the German population. The WIdO is the Research Institute of the AOK and for example, it conducts health economic, epidemiological, and health services research. Enrollment to SHI is unrestricted regarding age, health status, income, employment, or geographical region. The study data included information on insured persons with DS, identified by the ICD-10-GM (International Classification of Diseases—German Modification) three-digit Q90.- (Down syndrome), as well as those without DS. Insured persons were only included if they had been insured with the respective health insurance fund for a continuous period of at least one year and had been resident in Germany. The data included information on sex, age groups, and ICD-10-GM-Codes for (Alzheimer's) dementia and DS. (Alzheimer's) dementia diagnoses from the inpatient and outpatient hospital sectors and diagnoses from SHI-accredited physicians were used, with different validation criteria used for each sector due to differences in coding quality (see Empirical Approach). In the German remuneration system of in- and outpatient medical care, the coding of diagnoses is mandatory for remuneration. According to Section 295 of the German Social Code, Book V (Fünftes Buch Sozialgesetzbuch—SGB V), physicians and institutions participating in SHI are obliged to code diagnoses for medical treatment. For this reason, it can be assumed that uncodified (Alzheimer's) dementia diagnoses were also not diagnosed. Due to data protection reasons, only group sizes of at least 10 people may be specified in the analysis. The study was approved by the ethics committee of the medical faculty of the University of Duisburg-Essen (ref. no. 22-10854-BO, 10 Aug 2022).

### Empirical approach

Several validation steps, which differed slightly between the two groups of those with and without DS, are described in detail below and were used to identify insured persons with (Alzheimer's) dementia.

**Figure 1.**
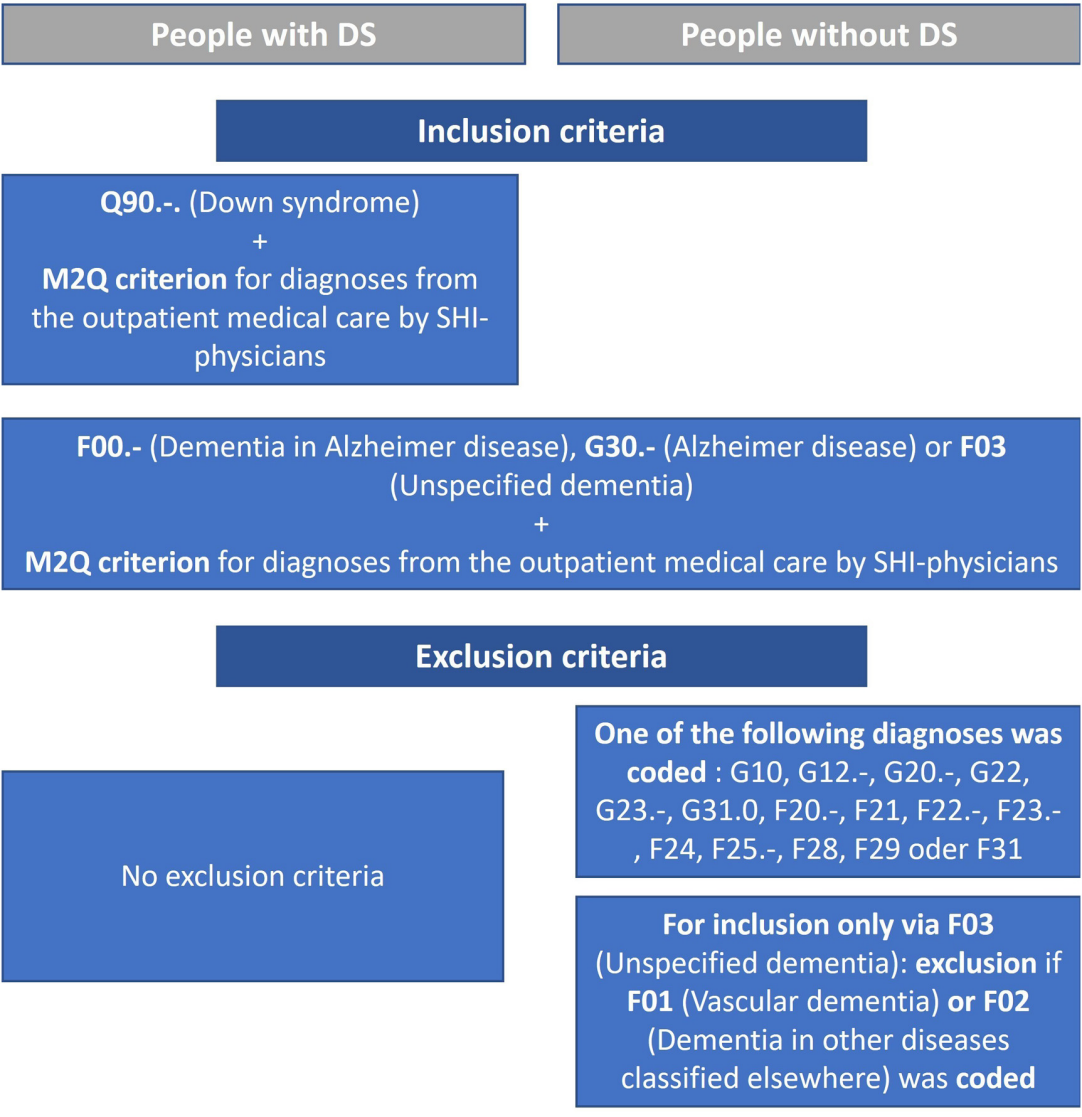
Inclusion and exclusion criteria.

In both groups (adults with and without DS), insured persons had to have at least one diagnosis coded according to ICD-10-GM three-digit F00.- (Dementia in AD), G30.- (AD), or F03 (Unspecified dementia) (see Figure 1). Unspecific dementia diagnoses were considered, as specific coding is often not utilized in medical practice.^29,30^ To reduce the risk of including misdiagnoses, it is standard practice in German claims-based research to require at least two confirmed outpatient diagnoses documented in different quarters within one year (M2Q criterion^31^). This approach is widely used in studies in Germany (e.g.,^32^) and is also implemented in the morbidity-oriented risk adjustment scheme (Morbi-RSA) of the German SHI, where outpatient diagnoses are considered valid only if the M2Q criterion is fulfilled.^33^ To avoid excluding people whose diagnoses span the end of a calendar year, the dynamic M2Q criterion was applied, meaning that the respective diagnosis had to be coded in at least two out of four consecutive quarters. For example, diagnoses documented in the fourth quarter of one year and the first quarter of the following year would meet the criterion. In the hospital setting, main and secondary diagnoses were taken into account. In the population without DS, people had already been preselected by the WIdO. In the interests of data minimization, only the preselected results were transmitted to the evaluator.

Strict additional exclusion criteria were applied to adults without DS in order to ensure that the study population primarily comprised individuals with a clear diagnosis of AD and no other forms of dementia. This was necessary because, in contrast to adults with DS who have a genetic predisposition to AD, adults without DS do not have a comparable, uniform genetic predisposition. In the general population, dementia diagnoses are etiologically more heterogeneous and may reflect a wide range of primary and secondary causes. For this reason, additional exclusion criteria were applied in the non-DS population to ensure that identified cases primarily represent AD, whereas applying the same exclusions in the DS population would not be appropriate and could introduce selection bias. The comparison group (population without DS) should assess whether the possible underdiagnosis is specific to the DS population. To exclude persons who actually suffer from a diagnosis of the differential diagnoses of dementia, persons with the following diseases were not included: Parkinson disease, Huntington disease, spinal muscular atrophy and related syndromes, other degenerative diseases of the basal ganglia, circumscribed brain atrophy, Schizophrenia, Schizotypal disorder, persistent delusional disorders, acute and transient psychotic disorders, induced delusional disorder, Schizoaffective disorders, other nonorganic psychotic disorders, unspecified nonorganic psychosis and bipolar affective disorder. If only the non-specific dementia code F03 (unspecified dementia) and none of the AD-specific codes (F00, G30) were present, an additional exclusion was made if there was also a diagnosis of vascular dementia (F01) or dementia due to another underlying disease (F02).

### Statistical analysis

In this study, the administrative prevalence is calculated using the quotient of the number of diagnosed adults with (Alzheimer's) dementia in the respective population (Population 1: Adults with DS, Population 2: Adults without DS) and the number of all individuals in the corresponding population in the respective data year.

The analyses were conducted as a function of age and sex for every data year, with age treated as a time-varying variable. In the population of adults with DS, the administrative prevalence by coded type of dementia was also examined. The background to this analysis was primarily an investigation of coding quality, as AD is expected to be highly prevalent in the group of adults with DS. For all prevalence analyses, mean values over the entire observation period (2010 to 2019) and the standard deviation were calculated.

Results were considered statistically significant if p < 0.05. P-values were calculated using the Chi-Square test^34^ because only categorical variables are included in the analyses. Cramér's V^35^ was calculated to show the strength of a relationship between variables (e.g., age group) within the respective groups (adults with DS or adults without DS). For two dichotomous characteristics, Phi was used as the measure of association. According to Cohen, effect sizes that are smaller than the value |0.1| are considered irrelevant, above |0.1| weak, above |0.3| medium and above |0.5| large.^36^ For differences between the two populations, Cramér's V and Phi were not used because the values would be distorted by the very large differences in group size between the two populations. Instead, the odds ratios (ORs)^37^ were calculated by using a contingency table to compare the two populations for every data year. To analyze group-specific associations over time, the Mantel-Haenszel odds ratio (OR_MH_)^38^ was calculated. The 95% confidence intervals were also calculated for the ORs.^39^

## Results

### Study population

Table 1 shows the mean values (MV) and standard deviations (SD) of the populations with and without DS by sex and age group during the observation period (2010–2019). In the population without DS, the mean proportion of males was 47.9%, and 69.3% of individuals were aged 31 years or older. In the population with DS, the mean proportion of males during the observation period was 45.5%, and similarly, the majority of persons were aged 31 years or older (MV 64.9%).

**Table 1.** Mean values and standard deviations of the populations of insured persons with and without DS by age group and sex during the observation period (2010–2019).

	**Persons without DS**		**Persons with DS**	
	**Quantity**	**in %**	**Quantity**	**in %**
**Sex**				
male	11,449,280 (± 537,073)	47.9 (± 0.6)	14,398 (± 51)	45.5 (± 0.2)
female*	12,456,625 (± 312,577)	52.1 (± 0.6)	17,216 (± 165)	54.5 (± 0.2)
**Age group (in years)**				
≤30	7,340,585 (± 423,957)	30.7 (± 0.7)	11,090 (± 130)	35.1 (± 0.5)
31–35	1,343,315 (± 206,455)	5.6 (± 0.7)	2758 (± 83)	8.7 (± 0.3)
36–40	1,278,628 (± 139,240)	5.3 (± 0.4)	2977 (± 87)	9.4 (± 0.3)
41–45	1,438,965 (± 108,341)	6.0 (± 0.6)	3266 (± 303)	10.3 (± 1.0)
46–50	1,733,621 (± 69,703)	7.3 (± 0.5)	3518 (± 157)	11.1 (± 0.5)
51–55	1,807,320 (± 114,851)	7.6 (± 0.3)	3122 (± 254)	9.9 (± 0.8)
56–60	1,647,600 (± 147,400)	6.9 (± 0.4)	2309 (± 164)	7.3 (± 0.5)
61–65	1,436,635 (± 114,110)	6.0 (± 0.3)	1411 (± 163)	4.5 (± 0.5)
66–70	1,257,692 (± 119,578)	5.3 (± 0.5)	618 (± 129)	2.0 (± 0.4)
≥71	4,621,545 (± 183,546)	19.0 (± 1.4)	546 (± 49)	1.7 (± 0.1)
71–75	1,357,615 (± 283,711)	5.7 (± 1.4)	For data protection reasons, no differentiation is possible here in the population with DS.	
76–80	1,375,706 (± 74,339)	5.8 (± 0.4)		
81–85	1,018,329 (± 52,967)	4.3 (± 0.1)		
86–90	592,263 (± 25,397)	2.5 (± 0.1)		
≥91	277,632 (± 33,086)	1.2 (± 0.1)		

* For data protection reasons and in line with the standard procedure in SHI, persons with the sex “diverse” were assigned to the female category.

### Total administrative prevalence

**Figure 2.**
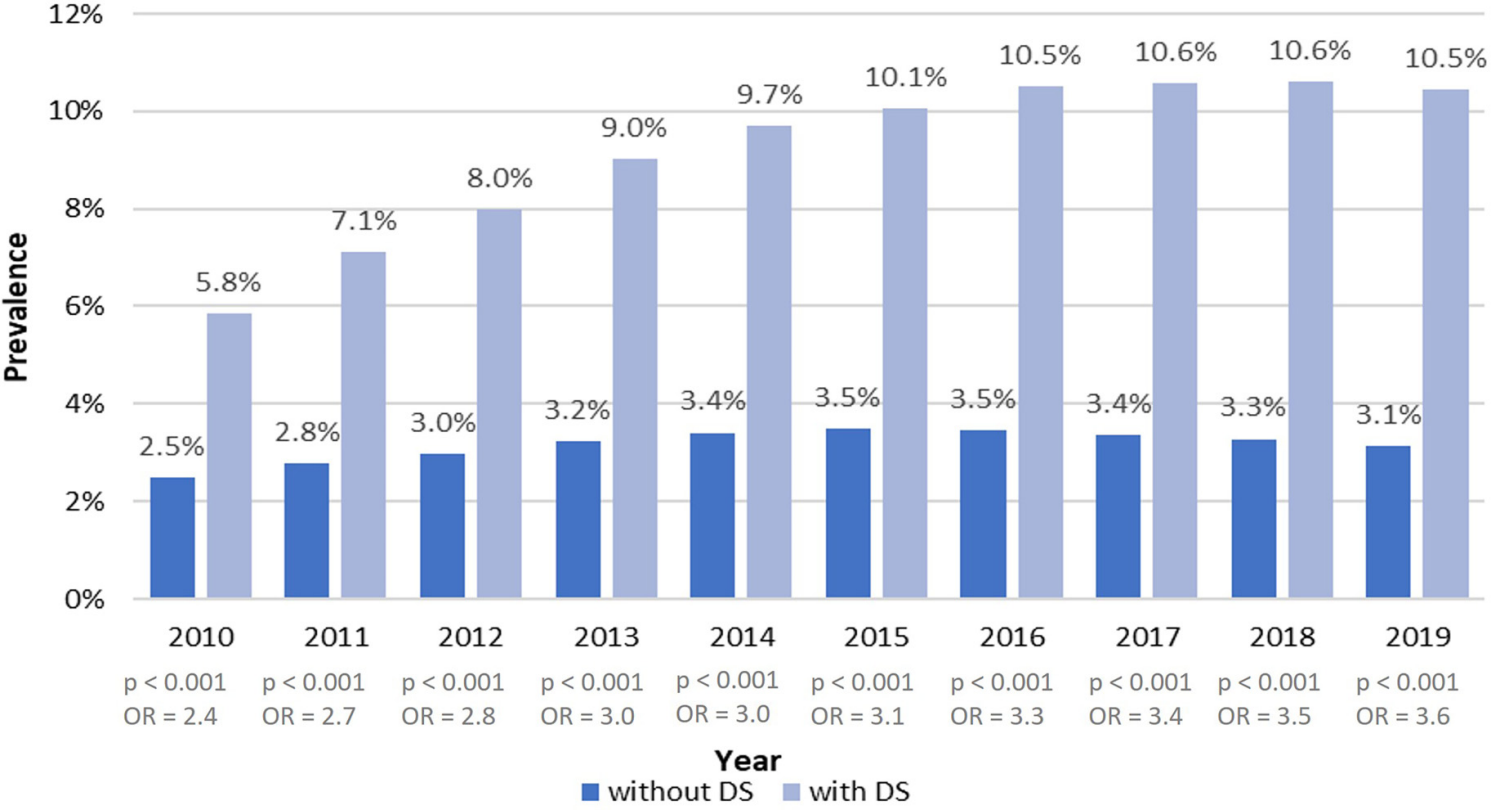
(Alzheimer's) dementia administrative prevalence (total).

Figure 2 shows the administrative prevalence of (Alzheimer's) dementia during the observation period (2010–2019), separately for the population with DS (MV 9.2% ± 1.7% (SD)) and the population without DS (MV 3.2% ± 0.3% (SD)). The difference in administrative prevalence between the two populations is significant (p < 0.001) for each year. The administrative prevalence increased over the observation period in the DS population, while remaining relatively consistent in the non-DS population.

OR: odds ratio.

The OR of administrative dementia prevalence for the coded diagnoses is 2.4 in 2010 and 3.6 in 2019, meaning that adults with DS were 2.4-times more likely to be coded with an (Alzheimer's) dementia in 2010 and 3.6-times in 2019 than adults without DS. The OR_MH_ was 3.1 [95% CI: 3.0–3.2] across all years.

### Administrative prevalence by age groups

When looking at administrative prevalence as a function of age, large differences between the two populations are noticeable. Table 2 presents mean annual prevalence and corresponding SD by age group across the analysis period (2010–2019), rather than person-level values. The results for individual years can be found in Supplemental Table 1. The administrative prevalence in the population of adults with DS in each age group was much higher than in the comparison group (e.g., in the age group from 56 to 60 years: MV 28.7% ± 4.6% (SD) versus MV 0.7% ± 0.1% (SD)). In addition to the higher administrative prevalence of (Alzheimer's) dementia in all age groups, an earlier onset of (Alzheimer's) dementia was observed in the population with DS. It can also be seen that the administrative prevalence of (Alzheimer's) dementia in the population with DS increased up to the age group of 66–70 years (MV 39.9% ± 6.7% (SD)), but decreased in the age group of over 70 years (MV 26.1% ± 9.8% (SD)).

**Table 2.** Mean values and standard deviations of the administrative (Alzheimer's) dementia prevalence (adults with and without DS) by age group during the observation period (2010–2019), as well as the OR_MH_.

Age group in years	<30	31–35	36–40	41–45	46–50	51–55	56–60	61–65	66–70	≥ 71
	MV (± SD)	MV (± SD)	MV (± SD)	MV (± SD)	MV (± SD)	MV (± SD)	MV (± SD)	MV (± SD)	MV (± SD)	MV (± SD)
**With DS** in %	0.4 (± 0.1)	2.1 (± 0.3)	2.9 (± 0.4)	5.3 (± 0.4)	10.8 (± 1.3)	18.8 (± 2.5)	28.7 (± 4.6)	35.1 (± 5.1)	39.9 (± 6.7)	26.1 (± 9.8)
**Without DS** in %	0.0 (± 0.0)	0.1 (±0.0)	0.1 (± 0.0)	0.2 (± 0.0)	0.2 (± 0.0)	0.4 (± 0.1)	0.7 (± 0.1)	1.3 (± 0.2)	2.4 (± 0.3)	14.4 (± 2.1)
**OR_MH_**	19.0	25.8	28.8	36.7	51.6	59.2	57.8	44.2	28.3	2.1
**95% CI [OR_MH_]**	12.7–28.5	16.9–39.4	20.0–41.5	27.5–49.1	40.9–65.0	48.6–72.2	48.1–69.6	44.1–44.2	22.8–35.0	1.9–2.3

MV: mean value; SD: standard deviation; OR_MH_: Mantel-Haenszel odds ratio; 95% CI: 95% confidence interval.

As was to be expected from the different age structure in the two populations, the difference in administrative prevalence between the two populations is significant in all age groups for every year (in each case: p < 0.001), except for the age group of ≥ 71 years in 2010 and 2011. The OR_MH_ ranges from 2.1 [95% CI: 1.9–2.3] to 59.2 [95% CI: 48.6–72.2], which indicates that adults with DS in this dataset are 2.1 (≥ 71 years) to 59.2-fold (51–55 years) more likely to be coded with (Alzheimer's) dementia than adults without DS.

The analysis also reveals differences depending on age within the respective population (p < 0.001 in each population for each year of observation). As expected with the very large proportion of people in the ≥ 71 age group in the population without DS, the effect size in the said population is very strong (Cramér's V = 0.95 to 0.96 in the different years). In the population with DS there is more of a medium effect with a value of 0.29 to 0.31 during the reporting period.

### Administrative prevalence by sex

The average administrative dementia prevalence in the period from 2010 to 2019 in the population without DS was 2.2% ± 0.3% (SD) for males and 4.0% ± 0.4% (SD) for females, whereas in the population with DS these values were 9.8% ± 1.8% (SD) for males and 8.7% ± 1.6% (SD) for females. Thus, the administrative prevalence was higher in both males and females in the population with DS than in the population without DS. This difference in administrative prevalence between adults with and without DS is significant in all data years (p < 0.001) for both sexes. Males with DS are 4.9 [95% CI: 4.7–5.1] times more likely to be coded with (Alzheimer's) dementia than males without DS, while the OR_MH_ is 2.3 [95% CI: 2.2–2.3] in females with DS compared to those without DS.

In the population without DS, the prevalence of (Alzheimer's) dementia among females was significantly higher than that of the male population for all data years (p < 0.001). However, Phi is very small here (0.05 to 0.06 in the different data years). The difference in the population with DS is also significant for all data years (p < 0.001: 2011, 2012 and 2015 to 2019; p < 0.01: 2010, 2013 and 2014; see Supplemental Table 2). Here, however, the effect size is even lower with a value of 0.01 to 0.02 in the different data years.

### Prevalence and frequencies by coded type of dementia

Analyses depending on the type of dementia were carried out to evaluate the coding quality of dementia in the population with DS. The average proportion (2010–2019) of persons with a respective dementia diagnosis (Alzheimer's disease specific form of dementia: dementia in Alzheimer disease with early onset [F00.0 or G30.0], dementia in Alzheimer disease with late onset [F00.1 or G30.1], dementia in Alzheimer disease, atypical or mixed type [F00.2 or G30.8] and dementia in Alzheimer disease, unspecified [F00.9 or G30.9] or unspecific dementia [F03]) of all persons with at least one dementia diagnosis was calculated. When the methodological criteria are fulfilled, individuals may be assigned multiple dementia diagnoses. Consequently, the aggregated frequencies of the different dementia diagnosis categories exceed 100%. AD with late onset (MV 7.1% ± 0.6% SD) or atypical or mixed AD (MV 6.8% ± 1.5% SD) was seldom coded in individuals with dementia. The unspecified AD (MV 14.7% ± 0.9% SD) and early-onset AD (MV 20.7% ± 4.4% SD) were coded more frequently, while unspecified dementia was coded most frequently among adults with dementia (MV 83.6% ± 1.8% SD).

During the entire observation period, the frequencies of diagnosis F03 was higher than the ones of diagnosis F00. At the beginning of the observation period (in 2010), however, the difference in frequencies between the diagnoses was still much higher than in 2019 (see Supplemental Table 4). The frequencies of the diagnoses increased during the observation period. While 79.7% of adults with DS and (Alzheimer's) dementia were diagnosed with F03 in 2010, this proportion was 84.7% in 2019, meaning an increase of 6.3%. An even greater increase can be observed regarding the diagnosis F00.0/G30.0. In 2010, 12.6% of adults with DS and dementia received this diagnosis; this proportion was 26.1% in 2019. This shows that the proportion of adults with DS and (Alzheimer's) dementia diagnosed with F00.0/G30.0 has increased by around 107%.

Examining the prevalence of dementia in people with DS by the coded type of dementia (F00/G30 or F03), the mean prevalence for ICD-10 code F00/G30 over the entire observation period is only 3.5% ± 0.9% (SD), whereas for F03 it is 5.8% ± 0.8% (SD). Overall, however, the proportion of F00/G30 increased during the observation period. At the beginning of the period (in 2010), this ratio was approx. 30%, whereas by 2019 it had risen to 41.8% (see Table 3).

**Table 3.** Prevalence of people with the different types of dementia (only people with DS).

Year	Prevalence in %		Proportion of prevalence in %	
	F00/G30	F03	F00/G30	F03
**2010**	1.8	4.1	30.0	70.0
**2011**	2.4	4.7	33.7	66.3
**2012**	2.7	5.3	33.8	66.2
**2013**	3.1	5.9	34.5	65.5
**2014**	3.6	6.1	36.7	63.3
**2015**	3.8	6.3	37.2	62.3
**2016**	4.1	6.4	39.2	60.8
**2017**	4.2	6.3	40.2	59.8
**2018**	4.4	6.3	41.0	59.0
**2019**	4.4	6.1	41.8	58.2
**MV** **(± SD)**	3.5 (± 0.9)	5.8 (± 0.8)	36.9 (± 3.8)	63.1 (± 3.8)

MV: mean value; SD: standard deviation.

### Limitations

It should be emphasized that the analyzed claims data only include a large proportion of SHI-insured persons (approx. 37%), excluding approx. 10% with private health insurance in Germany.^40^ Furthermore, only data from the AOK were analyzed. Due to historical reasons, different health insurance funds cover populations that differ in age, sex, and socio-economic status, which limits the applicability of the results to the German population as a whole.^41,42^ Supplemental Table 5 compares the age and sex distributions of the study population and all SHI-insured persons, demonstrating a strong similarity between the two groups.

Claims data are primarily collected for billing purposes, which can sometimes lead to inadequate diagnosis documentation. For example, a non-specific diagnosis may be coded instead of a specific one. As claims data do not contain clinical information, it is not possible to provide information on aspects such as the severity of a disease. Moreover, miscoding or upcoding can occur, for which financial incentives can be responsible.^43^

It is important to distinguish between administrative prevalence derived from claims data and research-based clinical prevalence. Claims data reflect diagnosed cases in routine care and thus primarily capture healthcare utilization and diagnostic practices rather than the true underlying disease burden. Accordingly, the prevalence estimates reported here should be interpreted as indicators of potential underdiagnosis, not as measures of true (Alzheimer's) dementia prevalence.

The number of adults with DS diagnosed with dementia is a hidden variable and can only be approximated using the coded diagnosis frequency of dementia diagnoses. While physicians are legally obligated to document diagnosed illnesses in accordance with Section 295 of the German Social Code, Book V, it is possible that in individual cases, particularly in outpatient care, the diagnosis of (Alzheimer's) dementia is not recorded in a comprehensive or uniform manner. Validation steps such as the dynamic M2Q criterion were used to increase the certainty that the persons actually had (Alzheimer's) dementia. However, the application of the dynamic M2Q criterion could also lead to a slight bias in diagnosed (Alzheimer's) dementia illnesses, as it could be that patients who have only received one (Alzheimer's) dementia diagnosis within four quarters still have (Alzheimer's) dementia. Also, a (Alzheimer's) dementia diagnosis requires contact with a physician; people with infrequent visits may remain undiagnosed.

## Discussion

The present manuscript investigates, based on claims data, whether the prevalence of (Alzheimer's) dementia observed in epidemiological studies among people with DS is similarly reflected in standard care. Due to the genetic risk of adults with DS, epidemiological studies can be considered a reliable representation of the actual rates. Furthermore, in this manuscript, (Alzheimer's) dementia was examined in adults without DS to assess whether the observed underdiagnosis in the DS group is attributable to general diagnostic limitations or to challenges specific to individuals with DS.

Comparing administrative prevalence values with epidemiological prevalence values, the very low administrative values clearly indicate underdiagnosis among SHI-insured persons with DS in Germany. For example, Lai et al. (1989) reported a (Alzheimer's) dementia prevalence of 75% among adults with DS over the age of 60,^12^ whereas Tyrrell et al. (2001) found a significantly lower prevalence of 41.7% in the same age group.^18^ Among those aged 60–70, Prasher et al. (1995) reported a prevalence of 54.5%,^15^ while Visser et al. (1997) found an even higher rate of 77%.^13,15^ Notably, Visser et al. (1997) reported a (Alzheimer's) dementia prevalence of 100% among adults with DS over the age of 70.^13^ McCarron et al. identified a (Alzheimer's) dementia risk of 80% to 88% for individuals over 65 years of age.^10,14^ In our study, 35.1% (MV) of the age group 61–65 years received a(n) (Alzheimer's) dementia diagnosis and 39.9% (MV) in the age group 66–70 years; the group with the highest frequency of (Alzheimer's) dementia diagnoses. Although the administrative prevalence is still too low compared to the expected values, it increased in the observation period. The reasons for underdiagnosis can be categorized as follows. Firstly, there are barriers to access. For instance, there is a lack of awareness regarding the increased risk of AD in individuals with DS, which results in individuals with DS not seeking medical attention. In addition, there is a shortage of specialists able to provide the necessary diagnostic services.^44^ Secondly, there are challenges in carrying out (Alzheimer's) dementia diagnostics.^45,46^ One such challenge is the lack of knowledge about specific dementia screening procedures for people with intellectual disabilities in general or for adults with DS in particular among standard care providers,^47^ e.g., the DSQIID.^25^ Another challenge in diagnosis is that individuals with DS frequently exhibit concomitant health impairments, such as thyroid disease. (Alzheimer's) dementia symptoms may be mistakenly attributed to these impairments.^48^ As symptoms can present differently than in the general population, a considerable proportion with moderate to severe dementia may be excluded by applying ICD-10 criteria.^49^ As a result, as already described in the Limitations section, (Alzheimer's) dementia is likely underdiagnosed in claims-based datasets, which may explain the lower prevalence observed in our study compared with clinical cohorts.

In the DS population, administrative prevalence was slightly higher in a comparable U.S. study by Rubenstein et al. (13.7% in 2011 and 15.3% in 2019 versus 7.1% in 2011 and 10.5% in 2019 in our study).^50^ In a claims data analysis, Bayen et al. (2018) found a much higher prevalence in the different age groups; in their study, the maximum was 56% in the 66–65 age group.^51^ In our study, prevalence increased with age in the DS population up to the 66–70 group (MV 39.9% and 45.6% in 2018).

What is striking in the population with DS is that the prevalence decreases again in the age group of ≥ 71 years. Other studies have also shown a decrease in the prevalence of (Alzheimer's) dementia in older DS patients.^8,51^ This could be due to survival bias and diagnostic attrition. (Alzheimer's) dementia in individuals with DS typically has an early onset and is associated with increased mortality.^3,8^ As a result, people with DS and (Alzheimer's) dementia are less likely to survive into the oldest age groups, leading to a selective survival of less severely affected people.^50^ Additional analyses of the present study showed that approx. 50% of people with DS died within the first five years following the initial diagnosis of (Alzheimer's) dementia. Furthermore, for 44% of those whose first diagnosis occurred in 2010 or 2011, no documented date of death was recorded by the end of the observation period. Mortality therefore contributes to declining prevalence in the oldest age groups but does not fully explain the observed results. In addition, when relying on claims data rather than systematic clinical assessments, (Alzheimer's) dementia may be underdiagnosed in routine care, as cognitive decline may be attributed to other age-related conditions or comorbidities.^51^ Outpatient diagnoses may be attributed less frequently in older people^52^ because they are less likely to undergo specialist medical examinations,^53^ which are often required for (Alzheimer's) dementia diagnoses to be documented in claims data.

In the population without DS, average prevalence is 3.2% ± 0.3% (SD) and slightly higher compared to other studies,^54,55^ even though stricter criteria were applied in our study for the population without DS than for the population with DS. Also, the (Alzheimer's) dementia values reported by Lobo et al. (2000) for the individual age groups were slightly lower than those observed in our study (e.g., Lobo: 0.6% in the 65–69 age group; our study: 2.4% in the 66–70 age group).^56^ The findings indicate that underdiagnosis is not a general issue in the general population, but is particularly noticeable among adults with DS. This diagnostic discrepancy appears to be due to challenges specific to diagnosing (Alzheimer's) dementia in this group.

The increase in coded dementia over the years (Figure 2), especially in adults with DS, could be an indicator that relatives and (medical) professional groups are becoming increasingly aware of the genetically determined high risk of (Alzheimer's) dementia in this group of people. It may also be caused by the availability of screening procedures that support the diagnosis of (Alzheimer's) dementia in people with an intellectual impairment. A shift in the age structure, attributable to an increase in the proportion of older age groups in the DS population, can also influence the overall prevalence. Although the proportion of these groups is increasing slightly (see Supplemental Table 6), the increase in prevalence cannot be explained by this alone because an increase in prevalence was observed in all age groups from the 46–50 age group onwards in the population with DS. Changes in reimbursement incentives related to the coding of corresponding diagnoses could also be a reason for an increase in administrative prevalence. In the German SHI system, payment incentives for coding dementia exist in the chronic lump sums and special geriatric lump sums, which can be billed regardless of age in the presence of (Alzheimer's) dementia.^57^ The chronic care lump sums with its current structure and the geriatric lump sums were introduced in 2013.^58^ However, earlier versions of chronic lump sums already existed, so no entirely new incentive was created during the observation period. Only a slight rise in prevalence was seen afterwards, particularly in the population without DS. In the population with DS, the prevalence has continued to rise since 2013, but the increase in previous years is much stronger. Therefore, the observed increase in administrative prevalence cannot be primarily attributed to payment, although it may be a contributing factor to the coding behavior of physicians.^59^

What is noteworthy about the analyses as a function of sex is that more males in the group of adults with DS have dementia, while the sex ratio is the other way around in the group of adults without DS. The sex difference in the group without DS has been examined in several studies and is likely due to a combination of factors, including the higher life expectancy of women, which increases the risk of developing dementia, and hormonal changes caused by the decline in estrogen levels during menopause.^60,61^ The observed differences may not solely reflect actual biological differences in sex, but could also be influenced by differences in healthcare utilization. For instance, men and women have different patterns of healthcare utilization: women generally visit outpatient facilities more often,^62^ which increases the likelihood of cognitive symptoms being recognized and documented. Observed sex differences may also reflect biases in detection. Behavioral phenotypes vary by sex in the non-DS population.^63^ This may affect the recognition and coding of diagnoses in routine care.

The results of other studies on sex differences in adults with DS are mixed. Most found no difference by sex in adults with DS^8,50,51,64^ or a higher risk for women.^65–68^ Mhatre et al. found a six-fold higher risk in males over the age of 60.^69^ The authors cite hormonal abnormalities in testosterone production in men with DS, which progress with age, as a possible explanation. However, in further analyses of the present study examining sex differences by age, no differences were observed among adults with DS aged between 51 and 70 years for most of the years analyzed (see Supplemental Table 3). The administrative prevalence of (Alzheimer's) dementia in adults with DS may also be influenced by detection or coding biases, such as behavioral phenotypes by sex. However, Urv et al. (2008) found that psychiatric symptoms in adults with DS and (Alzheimer's) dementia occur independently of sex.^70^ A study by Ahlström et al. (2020) reported that women with DS were more likely than men to plan a physician's visit for dementia.^71^ Sex differences in adults with DS and AD, as well as potential contributing factors, need to be investigated further.

The analyses depending on the type of dementia showed that the diagnosis F03 (“Unspecified dementia”) is coded frequently in the population with DS and the prevalence of F03 in this population exceeds the prevalence of AD. As already described, almost all dementia cases in adults with DS should be attributable to AD due to the genetic predisposition. The diagnosis of AD requires certain criteria. There are different possible reasons for the low proportion of AD diagnoses. One reason could be that medical examinations, including imaging procedures, are more challenging to perform on people with DS or are considered less important, which is why non-specific dementia is coded instead. Other reasons for inaccurate coding may include a lack of time or difficulties with the coding process itself.^72–75^ Although AD is the most common cause of dementia in adults with DS, other causes may also contribute to cognitive decline. Studies have reported the presence of Lewy bodies^76,77^ and vascular changes^78^ in adults with DS and AD, suggesting that mixed pathological mechanisms may occur in this population. In addition, people with intellectual disability have a higher prevalence of mental illness compared to the general population.^79^ Just as symptoms of (Alzheimer's) dementia can be misinterpreted as psychiatric disorders, psychiatric symptoms can conversely be mistaken for (Alzheimer's) dementia-related changes. In routine clinical practice, etiological differentiation is often difficult, which may be a reason for nonspecific dementia diagnoses (ICD-10 F03). Consequently, some dementia cases captured under F03 in our data may reflect non-AD or mixed etiologies, which should be considered when interpreting prevalence estimates. However, given the strong biological predisposition to AD in DS and evidence from clinical and neuropathological studies indicating that AD pathology accounts for the majority of dementia cases in this population, we expect the overall impact of non-AD etiologies on our prevalence estimates to be limited.

In the context of the possibilities of Alzheimer's therapy at an early stage, a correct AD diagnosis could become increasingly relevant. The drug Lecanemab, licensed in the U.S. in July 2023,^80^ can reduce markers of amyloid in early AD.^81^ This way, the medication should help reducing the progression of the disease.^82^ In April 2025, the European Commission announced its decision to approve Lecanemab for the treatment of early-stage AD.^83^ Systemic underdiagnosis of AD in adults with DS could therefore substantially restrict their access to new treatments, despite the fact that this population has a well-researched genetic predisposition to AD. Nevertheless, it must be emphasized that the utilization of Lecanemb is currently not recommended for adults with DS due to insufficient data.^84^ There is only one study that has tested Lecanemab in adults with DS; the results show that Lecanemab may have potential in this population, but further studies are needed to investigate its efficacy and possible side effects.^85^

### Conclusion

Due to their genetic predisposition, adults with DS have a greatly increased risk of developing an early form of AD compared to the general population. Overall, however, a comparison of epidemiological prevalence rates with data from insurance claims has shown a potential underdiagnosis of (Alzheimer's) dementia in this group. Importantly, claims data primarily reflect healthcare utilization and diagnostic practices rather than the true underlying disease burden. The reasons for this are manifold and were researched in more detail in other parts of the “DS-Demenz” project.^44,47,86^ For example, despite the increasing availability of special screening instruments for people with intellectual impairments, these tools are not yet widely known or routinely implemented in practice. However, early diagnosis is highly relevant so that affected patients can receive appropriate care; this is particularly important in the context of a drug that could be used in the early stages of AD. Barriers and obstacles to the diagnosis of (Alzheimer's) dementia in adults with DS should continue to be researched so that these can be counteracted and the people affected receive an early diagnosis and the best possible treatment.

## Supplemental Material

sj-docx-1-alz-10.1177_13872877261449417 - Supplemental material for (Alzheimer's) dementia in adults with Down syndrome in Germany: Administrative prevalence based on a claims data analysis Supplemental material, sj-docx-1-alz-10.1177_13872877261449417 for (Alzheimer's) dementia in adults with Down syndrome in Germany: Administrative prevalence based on a claims data analysis by Milena Weitzel, Anke Walendzik, Godwin D. Giebel, Pascal Raszke, Jürgen Wasem, Johannes Levin, Georg Nübling, Olivia Wagemann, Elisabeth Wlasich, Johannes Pantel, Valentina A. Tesky, Arthur Schall, Thomas Ruhnke, Patrik Dröge and Theresa Hüer in Journal of Alzheimer's Disease
