# Supplementary material for: (Alzheimer's) dementia in adults with Down syndrome in Germany: Administrative prevalence based on a claims data analysis
Source: J Alzheimers Dis. 2026 May 15;112(1):161–74. doi: 10.1177/13872877261449417 (PMC13291392; doi:10.1177/13872877261449417)
Supplement: sj-docx-1-alz-10.1177_13872877261449417 - Supplemental material for (Alzheimer's) dementia in adults with Down syndrome in Germany: Administrative prevalence based on a claims data analysis [file sj-docx-1-alz-10.1177_13872877261449417.docx]

## **Supplemental Material**

**(Alzheimer's) dementia in adults with Down syndrome in Germany: Administrative prevalence based on a claims data analysis**

### **Supplemental Table 1. (Alzheimer’s) dementia prevalence by age groups in the different years**

#### Administrative prevalence of people with and without DS in the different age groups

| **Age group**  in years | **≤ 30** | | | | | **31-35** | | | | **36-40** | | | | **41-45** | | | | **46-50** | | | |
| --- | --- | --- | --- | --- | --- | --- | --- | --- | --- | --- | --- | --- | --- | --- | --- | --- | --- | --- | --- | --- | --- |
| **Year** | **P1**  in % | **P2**  in % | **p** | **OR_(MH)_** | **P1**  in % | | **P2**  in % | **p** | **OR_(MH)_** | **P1**  in % | **P2**  in % | **p** | **OR_(MH)_** | **P1**  in % | **P2**  in % | **p** | **OR_(MH)_** | **P1**  in % | **P2**  in % | **p** | **OR_(MH)_** |
| **2010** | 0.47 | 0.02 | <0.001 | 26.8 | 1.6 | | 0.06 | <0.001 | 14.1 | 2.1 | 0.1 | <0.001 | 24.9 | 4.7 | 0.1 | <0.001 | 40.9 | 8.1 | 0.2 | <0.001 | 48.2 |
| **2011** | 0.49 | 0.02 | <0.001 | 24.8 | 1.6 | | 0.07 | <0.001 | 24.5 | 2.7 | 0.1 | <0.001 | 28.5 | 4.9 | 0.1 | <0.001 | 39.7 | 9.6 | 0.2 | <0.001 | 53.2 |
| **2012** | 0.58 | 0.02 | <0.001 | 26.8 | 1.7 | | 0.07 | <0.001 | 24.4 | 2.6 | 0.1 | <0.001 | 26.9 | 5.4 | 0.1 | <0.001 | 37.1 | 10.6 | 0.2 | <0.001 | 55.0 |
| **2013** | 0.53 | 0.02 | <0.001 | 21.3 | 2.2 | | 0.08 | <0.001 | 27.6 | 2.9 | 0.1 | <0.001 | 27.6 | 5.7 | 0.2 | <0.001 | 37.6 | 11.6 | 0.2 | <0.001 | 53.7 |
| **2014** | 0.50 | 0.03 | <0.001 | 18.8 | 2.4 | | 0.09 | <0.001 | 27.2 | 3.1 | 0.1 | <0.001 | 27.6 | 6.1 | 0.2 | <0.001 | 36.7 | 11.9 | 0.3 | <0.001 | 51.8 |
| **2015** | 0.48 | 0.03 | <0.001 | 18.0 | 2.2 | | 0.09 | <0.001 | 24.6 | 3.5 | 0.1 | <0.001 | 31.8 | 5.3 | 0.2 | <0.001 | 32.1 | 12.1 | 0.3 | <0.001 | 52.2 |
| **2016** | 0.41 | 0.03 | <0.001 | 16.3 | 2.3 | | 0.09 | <0.001 | 28.0 | 3.3 | 0.1 | <0.001 | 29.4 | 5.6 | 0.2 | <0.001 | 35.3 | 11.7 | 0.3 | <0.001 | 51.0 |
| **2017** | 0.36 | 0.02 | <0.001 | 15.9 | 2.4 | | 0.08 | <0.001 | 31.0 | 2.9 | 0.1 | <0.001 | 27.7 | 5.5 | 0.2 | <0.001 | 35.0 | 11.2 | 0.3 | <0.001 | 50.2 |
| **2018** | 0.26 | 0.02 | <0.001 | 12.9 | 2.3 | | 0.08 | <0.001 | 31.0 | 3.0 | 0.1 | <0.001 | 30.6 | 5.2 | 0.2 | <0.001 | 36.8 | 10.8 | 0.2 | <0.001 | 50.7 |
| **2019** | 0.15 | 0.02 | <0.001 | 8.5 | 2.2 | | 0.07 | <0.001 | 31.9 | 3.1 | 0.1 | <0.001 | 33.2 | 4.9 | 0.1 | <0.001 | 36.9 | 10.1 | 0.2 | <0.001 | 49.2 |
| **MV**  **(± SD)** | 0.42  (± 0.13) | 0.02  (± 0.00) |  | 19.0 | 2.1  (± 0.3) | | 0.08  (± 0.01) |  | 25.8 | 2.9  (± 0.4) | 0.1  (± 0.0) |  | 28.8 | 5.3  (± 0.4) | 0.2  (± 0.0) |  | 36.7 | 10.8  (± 1.3) | 0.2  (± 0.0) |  | 51.6 |

| **Age group**  in years | **51-55** | | | | **56-60** | | | | **61-65** | | | | **66-70** | | | | **≥ 71** | | | | |
| --- | --- | --- | --- | --- | --- | --- | --- | --- | --- | --- | --- | --- | --- | --- | --- | --- | --- | --- | --- | --- | --- |
| **Year** | **P1**  in % | **P2**  in % | **p** | **OR_(MH)_** | **P1**  in % | **P2**  in % | **p** | **OR_(MH)_** | **P1**  in % | **P2**  in % | **p** | **OR_(MH)_** | **P1**  in % | **P2**  in % | **p** | **OR_(MH)_** | | **P1**  in % | **P2**  in % | **p** | **OR_(MH)_** |
| **2010** | 13.5 | 0.3 | <0.001 | 53.7 | 19.7 | 0.5 | <0.001 | 47.6 | 23.9 | 0.9 | <0.001 | 36.3 | 25.9 | 1.8 | <0.001 | 19.5 | | 8.1 | 10.9 | n.s. | 0.7 |
| **2011** | 15.8 | 0.3 | <0.001 | 58.0 | 23.5 | 0.6 | <0.001 | 52.3 | 29.1 | 1.0 | <0.001 | 41.4 | 34.3 | 2.0 | <0.001 | 20.1 | | 14.7 | 12.2 | n.s. | 1.2 |
| **2012** | 17.2 | 0.4 | <0.001 | 58.9 | 25.2 | 0.6 | <0.001 | 52.4 | 32.1 | 1.1 | <0.001 | 43.2 | 34.9 | 2.1 | <0.001 | 24.5 | | 19.6 | 12.9 | <0.001 | 1.6 |
| **2013** | 19.2 | 0.4 | <0.001 | 59.8 | 28.0 | 0.7 | <0.001 | 53.2 | 36.7 | 1.3 | <0.001 | 45.7 | 36.6 | 2.4 | <0.001 | 23.5 | | 23.6 | 14.2 | <0.001 | 1.9 |
| **2014** | 19.8 | 0.4 | <0.001 | 56.1 | 28.9 | 0.8 | <0.001 | 51.7 | 38.7 | 1.4 | <0.001 | 45.1 | 43.3 | 2.6 | <0.001 | 29.0 | | 26.3 | 15.2 | <0.001 | 2.0 |
| **2015** | 19.5 | 0.5 | <0.001 | 53.9 | 32.1 | 0.8 | <0.001 | 58.7 | 37.8 | 1.4 | <0.001 | 42.2 | 42.7 | 2.6 | <0.001 | 28.2 | | 28.4 | 15.9 | <0.001 | 2.1 |
| **2016** | 20.8 | 0.4 | <0.001 | 58.8 | 32.4 | 0.8 | <0.001 | 60.2 | 39.0 | 1.4 | <0.001 | 44.2 | 45.5 | 2.6 | <0.001 | 31.6 | | 31.1 | 16.5 | <0.001 | 2.3 |
| **2017** | 21.0 | 0.4 | <0.001 | 61.7 | 32.9 | 0.8 | <0.001 | 63.8 | 38.5 | 1.4 | <0.001 | 44.0 | 45.1 | 2.6 | <0.001 | 32.3 | | 35.2 | 16.7 | <0.001 | 2.7 |
| **2018** | 21.0 | 0.4 | <0.001 | 64.0 | 33.0 | 0.7 | <0.001 | 67.3 | 38.3 | 1.4 | <0.001 | 44.6 | 45.6 | 2.5 | <0.001 | 32.5 | | 36.7 | 16.8 | <0.001 | 2.9 |
| **2019** | 20.5 | 0.4 | <0.001 | 65.8 | 31.4 | 0.7 | <0.001 | 65.9 | 37.6 | 1.3 | <0.001 | 45.7 | 45.3 | 2.4 | <0.001 | 33.4 | | 37.5 | 12.8 | <0.001 | 3.1 |
| **MV**  **(± SD)** | 18.1  (± 2.5) | 0.4  (± 0.1) |  | 59.2 | 28.7  (± 4.6) | 0.7  (± 0.1) |  | 57.8 | 35.2  (± 5.1) | 1.3  (± 0.2) |  | 44.2 | 39.9  (± 6.7) | 2.4  (± 0.3) |  | 28.3 | | 26.1  (± 9.8) | 14.1  (± 2.1) |  | 2.1 |

*Differences within the population of people with DS as a function of age group*

| **Year** | **p** | **Cramér's V** |
| --- | --- | --- |
| **2010** | <0.001 | 0.31 |
| **2011** | <0.001 | 0.30 |
| **2012** | <0.001 | 0.30 |
| **2013** | <0.001 | 0.29 |
| **2014** | <0.001 | 0.29 |
| **2015** | <0.001 | 0.29 |
| **2016** | <0.001 | 0.29 |
| **2017** | <0.001 | 0.29 |
| **2018** | <0.001 | 0.29 |
| **2019** | <0.001 | 0.29 |

*Differences within the population of people without DS as a function of age group*

| **Year** | **p** | **Cramér's V** |
| --- | --- | --- |
| **2010** | <0.001 | 0.96 |
| **2011** | <0.001 | 0.96 |
| **2012** | <0.001 | 0.96 |
| **2013** | <0.001 | 0.95 |
| **2014** | <0.001 | 0.95 |
| **2015** | <0.001 | 0.95 |
| **2016** | <0.001 | 0.95 |
| **2017** | <0.001 | 0.95 |
| **2018** | <0.001 | 0.95 |
| **2019** | <0.001 | 0.96 |

### **Supplemental Table 2.** (Alzheimer’s) dementia prevalence by sex in the different years

| **Age group**  in years | **Male** | | | | **Female** | | | |
| --- | --- | --- | --- | --- | --- | --- | --- | --- |
| **Year** | **With DS**  in % | **Without DS**  in % | **p** | **OR_(MH)_** | **With DS**  in % | **Without DS**  in % | **p** | **OR_(MH)_** |
| **2010** | 6.3 | 1.6 | <0.001 | 4.2 | 5.5 | 3.3 | <0.001 | 1.7 |
| **2011** | 7.6 | 1.8 | <0.001 | 4.5 | 6.7 | 3.6 | <0.001 | 1.9 |
| **2012** | 8.6 | 2.0 | <0.001 | 4.7 | 7.5 | 3.9 | <0.001 | 2.0 |
| **2013** | 9.5 | 2.2 | <0.001 | 4.7 | 8.6 | 4.1 | <0.001 | 2.2 |
| **2014** | 10.3 | 2.4 | <0.001 | 4.7 | 9.2 | 4.4 | <0.001 | 2.2 |
| **2015** | 10.7 | 2.5 | <0.001 | 4.7 | 9.5 | 4.4 | <0.001 | 2.3 |
| **2016** | 11.2 | 2.5 | <0.001 | 5.0 | 9.9 | 4.3 | <0.001 | 2.4 |
| **2017** | 11.3 | 2.4 | <0.001 | 5.1 | 10.0 | 4.3 | <0.001 | 2.5 |
| **2018** | 11.3 | 2.4 | <0.001 | 5.3 | 10.0 | 4.1 | <0.001 | 2.6 |
| **2019** | 11.3 | 2.3 | <0.001 | 5.5 | 9.8 | 3.9 | <0.001 | 2.7 |
| **MV**  **(± SD)** | 9.8  (± 1.8) | 2.2  (± 0.3) |  | 4.9 | 8.7  (± 1.6) | 4.0  (± 0.4) |  | 2.3 |

####

#### Differences within the population of people with DS as a function of sex

| **Year** | **p** | **Phi** |
| --- | --- | --- |
| **2010** | <0.01 | 0.02 |
| **2011** | <0.001 | 0.01 |
| **2012** | <0.001 | 0.02 |
| **2013** | <0.01 | 0.01 |
| **2014** | <0.01 | 0.02 |
| **2015** | <0.001 | 0.02 |
| **2016** | <0.001 | 0.02 |
| **2017** | <0.001 | 0.02 |
| **2018** | <0.001 | 0.02 |
| **2019** | <0.001 | 0.02 |

#### Differences within the population of people without DS as a function of sex

| **Year** | **p** | **Phi** |
| --- | --- | --- |
| **2010** | <0.001 | 0.06 |
| **2011** | <0.001 | 0.06 |
| **2012** | <0.001 | 0.06 |
| **2013** | <0.001 | 0.05 |
| **2014** | <0.001 | 0.05 |
| **2015** | <0.001 | 0.05 |
| **2016** | <0.001 | 0.05 |
| **2017** | <0.001 | 0.05 |
| **2018** | <0.001 | 0.05 |
| **2019** | <0.001 | 0.05 |

### **Supplemental Table 3.** Administrative prevalence by age group according to sex in the different years (only people with DS)

| **Age group**  in years | **≤ 30** | | | | **31-35** | | | | **36-40** | | | | **41-45** | | | | **46-50** | | | |
| --- | --- | --- | --- | --- | --- | --- | --- | --- | --- | --- | --- | --- | --- | --- | --- | --- | --- | --- | --- | --- |
| **Year** | **Male**  in % | **Female**  in % | **p** | **Phi** | **Male**  in % | **Female**  in % | **p** | **Phi** | **Male**  in % | **Female**  in % | **p** | **Phi** | **Male**  in % | **Female**  in % | **p** | **Phi** | **Male**  in % | **Female**  in % | **p** | **Phi** |
| **2010** | 0.53 | 0.43 | n.s. | - | 2.0 | 1.4 | n.s. | - | 2.5 | 1.9 | n.s. | - | 4.5 | 4.8 | n.s. | - | 8.3 | 7.8 | n.s. | - |
| **2011** | 0.52 | 0.46 | n.s. | - | 2.4 | 1.3 | n.s | - | 3.5 | 2.1 | <0.05 | 0.04 | 3.5 | 4.7 | n.s. | - | 9.3 | 9.9 | n.s. | - |
| **2012** | 0.59 | 0.56 | n.s. | - | 2.8 | 1.2 | <0.01 | 0.06 | 3.5 | 2.0 | n.s. | - | 5.9 | 4.9 | n.s. | - | 9.8 | 11.5 | n.s. | - |
| **2013** | 0.57 | 0.48 | n.s. | - | 3.1 | 1.7 | <0.05 | 0.04 | 4.0 | 2.3 | <0.05 | 0.05 | 6.5 | 5.0 | n.s. | - | 10.2 | 13.1 | <0.01 | 0.05 |
| **2014** | 0.57 | 0.44 | n.s. | - | 3.3 | 1.9 | <0.05 | 0.04 | 4.2 | 2.5 | <0.05 | 0.05 | 7.5 | 4.9 | <0.01 | 0.05 | 10.5 | 13.5 | <0.01 | 0.05 |
| **2015** | 0.54 | 0.41 | n.s. | - | 2.8 | 1.9 | n.s. | - | 4.8 | 2.9 | <0.01 | 0.05 | 6.7 | 4.3 | <0.01 | 0.05 | 11.5 | 12.7 | n.s. | - |
| **2016** | 0.50 | 0.33 | n.s. | - | 2.7 | 2.1 | n.s. | - | 4.8 | 2.5 | <0.01 | 0.06 | 7.3 | 4.5 | <0.01 | 0.06 | 12.1 | 11.4 | n.s. | - |
| **2017** | 0.45 | 0.27 | n.s. | - | 2.5 | 2.4 | n.s. | - | 4.8 | 2.0 | <0.001 | 0.08 | 7.8 | 4.0 | <0.001 | 0.08 | 12.0 | 10.4 | n.s. | - |
| **2018** | 0.26 | 0.26 | n.s. | - | 3.0 | 1.9 | n.s. | - | 4.6 | 2.2 | <0.01 | 0.07 | 7.3 | 4.1 | <0.001 | 0.07 | 12.2 | 9.7 | <0.05 | 0.04 |
| **2019** | 0.19 | 0.11 | n.s. | - | 2.9 | 1.8 | n.s. | - | 4.5 | 2.4 | <0.01 | 0.06 | 6.7 | 3.9 | <0.01 | 0.06 | 12.0 | 8.7 | <0.01 | 0.05 |
| **MV**  **(± SD)** | 0.47  (± 0.1) | 0.40  (± 0.1) | - | - | 2.7  (± 0.4) | 1.8  (± 0.4) | - | - | 4.1  (± 0.8) | 2.3  (± 0.3) | - | - | 6.5  (± 1.1) | 4.5  (± 0.4) | - | - | 10.8  (± 1.4) | 10.9  (± 1.9) | - | - |

| **Age group**  in years | **51-55** | | | | **56-60** | | | | **61-65** | | | | **66-70** | | | | **≥ 71** | | | |
| --- | --- | --- | --- | --- | --- | --- | --- | --- | --- | --- | --- | --- | --- | --- | --- | --- | --- | --- | --- | --- |
| **Year** | **Male**  in % | **Female**  in % | **p** | **Phi** | **Male**  in % | **Female**  in % | **p** | **Phi** | **Male**  in % | **Female**  in % | **p** | **Phi** | **Male**  in % | **Female**  in % | **p** | **Phi** | **Male**  in % | **Female**  in % | **p** | **Phi** |
| **2010** | 13.4 | 13.6 | n.s. | - | 19.6 | 19.7 | n.s. | - | 26.0 | 21.7 | n.s. | - | 26.9 | 25.2 | n.s. | - | 9.3 | 7.5 | n.s. | - |
| **2011** | 15.0 | 16.7 | n.s. | - | 24.9 | 22.1 | n.s. | - | 30.0 | 28.1 | n.s. | - | 36.5 | 32.7 | n.s. | - | 17.1 | 13.2 | n.s. | - |
| **2012** | 16.9 | 17.5 | n.s. | - | 26.6 | 24.0 | n.s. | - | 32.1 | 31.9 | n.s. | - | 41.6 | 29.2 | <0.01 | 0.13 | 21.7 | 18.3 | n.s. | - |
| **2013** | 18.7 | 19.6 | <0.01 | 0.05 | 27.5 | 28.4 | n.s. | - | 36.9 | 36.3 | n.s. | - | 43.0 | 31.3 | <0.01 | 0.12 | 25.9 | 22.0 | n.s. | - |
| **2014** | 19.5 | 20.0 | n.s. | - | 28.9 | 28.8 | n.s. | - | 38.2 | 39.1 | n.s. | - | 49.4 | 38.3 | <0.05 | 0.11 | 29.3 | 24.2 | n.s. | - |
| **2015** | 19.2 | 19.8 | n.s. | - | 31.8 | 32.3 | n.s. | - | 38.5 | 37.1 | n.s. | - | 47.5 | 39.0 | <0.05 | 0.08 | 30.2 | 26.9 | n.s. | - |
| **2016** | 20.5 | 21.1 | n.s. | - | 31.7 | 33.1 | n.s. | - | 40.9 | 37.4 | n.s. | - | 47.2 | 44.2 | n.s. | - | 35.6 | 28.2 | n.s. | - |
| **2017** | 20.1 | 21.7 | n.s. | - | 32.1 | 33.4 | n.s. | - | 39.6 | 37.6 | n.s. | - | 45.3 | 44.9 | n.s. | - | 42.2 | 31.1 | <0.001 | 0.11 |
| **2018** | 20.0 | 21.9 | n.s. | - | 33.7 | 32.3 | n.s. | - | 37.9 | 38.8 | n.s. | - | 48.1 | 43.9 | n.s. | - | 43.8 | 32.5 | <0.01 | 0.11 |
| **2019** | 19.2 | 21.7 | n.s. | - | 32.5 | 30.2 | n.s. | - | 39.8 | 35.6 | n.s. | - | 47.4 | 43.7 | n.s. | - | 44.6 | 33.4 | <0.01 | 0.11 |
| **MV** | 18.3  (± 2.4) | 19.4  (± 2.7) | - | - | 28.9  (± 4.4) | 28.4  (± 4.9) | - | - | 36.0  (± 4.9) | 34.4  (± 5.5) | - | - | 43.3  (± 6.9) | 37.3  (± 7.2) | - | - | 30.0  (± 11.9) | 23.9  (± 8.7) | - | - |

### **Supplemental Table 4.** Proportion of people with the different types of dementia out of all people with a dementia diagnosis in the different years (only people with DS)

| **Type of dementia** | **2010** | **2011** | **2012** | **2013** | **2014** | **2015** | **2016** | **2017** | **2018** | **2019** | **MV (± SD)** |
| --- | --- | --- | --- | --- | --- | --- | --- | --- | --- | --- | --- |
| **F00.0 oder G30.0**  in % | 12.6 | 15.7 | 17.5 | 19.4 | 21.0 | 21.7 | 23.1 | 24.1 | 25.4 | 26.1 | 20.7 (± 4.4) |
| **F00.1 oder G30.1**  in % | 5.6 | 6.9 | 7.6 | 7.0 | 7.1 | 7.2 | 7.4 | 7.4 | 7.1 | 7.4 | 7.1 (± 0.6) |
| **F00.2 oder G30.8**  in % | 4.2 | 4.8 | 5.7 | 6.8 | 6.9 | 7.1 | 7.5 | 8.3 | 8.3 | 8.5 | 6.8 (± 1.5) |
| **F00.9 oder G30.9**  in % | 13.1 | 14.4 | 13.7 | 13.9 | 15.0 | 15.4 | 15.5 | 15.4 | 15.2 | 15.6 | 14.7 (± 0.9) |
| **F03**  in % | 79.7 | 81.1 | 82.8 | 84.7 | 84.7 | 84.6 | 84.4 | 84.5 | 85.1 | 84.7 | 83.6 (± 1.8) |

### **Supplemental Table 5.** Information on age and sex of SHI-insured persons and study population (2019)

|  | **SHI-insured persons in Germany** | | **Study population** | |
| --- | --- | --- | --- | --- |
|  | **Quantity** | **Percent** | **Quantity** | **Percent** |
| **Sex** | | | | |
| male | 35,115,287 | 48.1% | 12,274,712 | 48.6% |
| female* | 37,893,950 | 51.9% | 12,997,056 | 51.4% |
| **Age group (in years)** | | | | |
| ≤ 30 | 21,539,593 | 29.5% | 7,960,859 | 31.5% |
| 31-35 | 4,963,146 | 6.8% | 1,667,648 | 6.6% |
| 36-40 | 4,761,863 | 6.5% | 1,526,801 | 6.0% |
| 41-45 | 4,363,849 | 6.0% | 1,380,215 | 5.5% |
| 46-50 | 4,588,395 | 6.3% | 1,594,071 | 6.3% |
| 51-55 | 5,855,676 | 8.0% | 1,913,517 | 7.6% |
| 56-60 | 5,901,040 | 8.1% | 1,901,099 | 7.5% |
| 61-65 | 4,870,318 | 6.7% | 1,589,893 | 6.3% |
| 66-70 | 4,174,974 | 5.7% | 1,406,828 | 5.6% |
| ≥ 71 | 11,990,383 | 16.4% | 4,330,837 | 17.1% |

* For data protection reasons and in line with the standard procedure in SHI, persons with the sex "diverse" were assigned to the female category.

### **Supplemental Table 6.** Information on age of insured persons with DS in the analyzed sample

|  | **2010** | | **2011** | | **2012** | | **2013** | | **2014** | | **2015** | | **2016** | | **2017** | | **2018** | | **2019** | |
| --- | --- | --- | --- | --- | --- | --- | --- | --- | --- | --- | --- | --- | --- | --- | --- | --- | --- | --- | --- | --- |
| **Age group** | **Quantity** | **in %** | **Quantity** | **in %** | **Quantity** | **in %** | **Quantity** | **in %** | **Quantity** | **in %** | **Quantity** | **in %** | **Quantity** | **in %** | **Quantity** | **in %** | **Quantity** | **in %** | **Quantity** | **in %** |
| **≤ 30** | 11,248 | 36.1 | 11,287 | 35.8 | 11,115 | 35.2 | 11,030 | 34.9 | 10,943 | 34.6 | 10,902 | 34.5 | 10,956 | 34.7 | 11,174 | 35.0 | 11,143 | 35.0 | 11,099 | 35.0 |
| **31-35** | 2,765 | 8.9 | 2,801 | 8.9 | 2,861 | 9.1 | 2,849 | 9.0 | 2,833 | 9.0 | 2,795 | 8.8 | 2,691 | 8.5 | 2,657 | 8.3 | 2,679 | 8.4 | 2,645 | 8.4 |
| **36-40** | 3,160 | 10.1 | 3,084 | 9.8 | 2,911 | 9.2 | 2,919 | 9.2 | 2,925 | 9.3 | 2,928 | 9.3 | 2,942 | 9.3 | 3,029 | 9.5 | 2,974 | 9.3 | 2,901 | 9.2 |
| **41-45** | 3,668 | 11.8 | 3,611 | 11.4 | 3,588 | 11.4 | 3,476 | 11.0 | 3,304 | 10.5 | 3,166 | 10.0 | 3,063 | 9.7 | 2,932 | 9.2 | 2,922 | 9.2 | 2,930 | 9.3 |
| **46-50** | 3,549 | 11.4 | 3,610 | 11.4 | 3,676 | 11.6 | 3,651 | 11.5 | 3,642 | 11.5 | 3,564 | 11.3 | 3,509 | 11.1 | 3,462 | 10.8 | 3,341 | 10.5 | 3,174 | 10.0 |
| **51-55** | 2,702 | 8.7 | 2,799 | 8.9 | 2,885 | 9.1 | 3,024 | 9.6 | 3,129 | 9.9 | 3,278 | 10.4 | 3,326 | 10.5 | 3,384 | 10.6 | 3,354 | 10.5 | 3,337 | 10.5 |
| **56-60** | 2,081 | 6.7 | 2,148 | 6.8 | 2,199 | 7.0 | 2,214 | 7.0 | 2,274 | 7.2 | 2,301 | 7.3 | 2,344 | 7.4 | 2,408 | 7.5 | 2,519 | 7.9 | 2,602 | 8.2 |
| **61-65** | 1,060 | 3.4 | 1,232 | 3.9 | 1,341 | 4.2 | 1,389 | 4.4 | 1,446 | 4.6 | 1,462 | 4.6 | 1,507 | 4.8 | 1,550 | 4.9 | 1,528 | 4.8 | 1,592 | 5.0 |
| **66-70** | 529 | 1.7 | 472 | 1.5 | 459 | 1.5 | 528 | 1.7 | 538 | 1.7 | 630 | 2.0 | 717 | 2.3 | 737 | 2.3 | 765 | 2.4 | 806 | 2.5 |
| **≥ 71** | 430 | 1.4 | 497 | 1.6 | 531 | 1.7 | 556 | 1.8 | 574 | 1.8 | 575 | 1.8 | 553 | 1.7 | 576 | 1.8 | 583 | 1.8 | 584 | 1.8 |
| **total** | 31,192 | 100.0 | 31,541 | 100.0 | 31,566 | 100.0 | 31,636 | 100.0 | 31,608 | 100.0 | 31,601 | 100.0 | 31,608 | 100.0 | 31,909 | 100.0 | 31,808 | 100.0 | 31,670 | 100.0 |
